# Pentraxin 3 as a Prognostic Biomarker in Patients with Systemic Inflammation or Infection

**DOI:** 10.1155/2014/421429

**Published:** 2014-11-03

**Authors:** Siguan Liu, Xin Qu, Feng Liu, Chunting Wang

**Affiliations:** ^1^Department of Critical Care Medicine, Provincial Hospital Affiliated to Shandong University, Jinan 250021, China; ^2^Department of Emergency, Zaozhuang Municipal Hospital, Zaozhuang, Shandong 277101, China; ^3^Department of Critical Care Medicine, Beijing Tiantan Hospital, Capital Medical University, Beijing 100050, China; ^4^Department of Internal Medicine, Rongcheng Traditional Hospital, Weihai 264300, China

## Abstract

*Purpose*. The long pentraxin 3 (PTX3) is a key component of the humoral arm of the innate immune system. PTX3 is produced locally in response to proinflammatory stimuli. We reviewed the usefulness of systemic levels of PTX3 in critically ill patients with systemic inflammatory response syndrome (SIRS), sepsis, and bacteremia, focusing on its diagnostic and prognostic value. *Methods*. A PubMed search on PTX3 was conducted. The list of papers was narrowed to original studies of critically ill patients. Eleven papers on original studies of critically ill patients that report on PTX3 in SIRS, sepsis, or bacteremia were identified. *Results*. Systematic levels of PTX3 have little diagnostic value in critically ill patients with SIRS, sepsis, or bacteremia. Systemic levels of PTX3, however, have superior prognostic power over other commonly used biological markers in these patients. Systemic levels of PTX3 correlate positively with markers of organ dysfunction and severity-of-disease classification system scores. Finally, systemic levels of PTX3 remain elevated in the acute phase and decreased on recovery. Notably, the age of the patients and underlying disease affect systemic levels of PTX3. *Conclusions*. The diagnostic value of PTX3 is low in patients with sepsis. Systemic levels of PTX3 have prognostic value and may add to prognostication of patients with SIRS or sepsis, complementing severity-of-disease classification systems and other biological markers.

## 1. Introduction

Pentraxin 3 is a secretory protein classified as a long pentraxin subfamily member of the pentraxin family. The pentraxin family proteins, which are evolutionarily conserved multimeric pattern recognition receptors and share a pentraxin-like domain in the C terminus, are recognized as key components of humoral innate immunity [1]. PTX3 has a unique 200-amino acid domain in its N terminus and is known to play multiple roles, including the regulation of inflammatory reaction, innate resistance to pathogens, and female fertility [2]. PTX3 is expressed in a variety of cells at inflammatory sites [3] and is also stored in neutrophil-specific granules [4]. As the pentraxin C-reactive protein (CRP) and serum amyloid P component (SAP) are well-known acute phase proteins, PTX3 may also be an acute phase biomarker. Recently, many studies on the circulating PTX3 level in clinical trials have been reported. These reports indicate that the PTX3 levels are significantly increased in tuberculosis, cardiovascular, kidney, and female reproductive system diseases, as well as severe traumatic brain injury [5–10]. In addition, systemic levels of PTX3 have been shown to have prognostic value in predicting the severity and outcome in patients with cancer [11, 12]. Elevated plasma levels of PTX3 were also found to have a strong prognostic value in dengue virus infection [13] and leptospirosis [14].

Systemic levels of PTX3 are also increased in critically ill patients [15]. However, the usefulness of PTX3 as a biological marker in critical illness is uncertain. The aim of this systematic review is to provide an overview of studies investigating the diagnostic and prognostic properties of PTX3 in critically ill patients with SIRS, sepsis, or bacteremia. We hypothesized that PTX3 has both diagnostic and prognostic values in these inflammatory conditions. We also hypothesized that systemic concentrations of PTX3 correlate with severity-of-disease classification system scores and other biological markers of severity of disease. Finally, we were interested in changes in systemic levels of PTX3 after initiation of treatment to observe whether PTX3 has any potential in guiding therapy.

## 2. Materials and Methods

### 2.1. Data Sources

Two methods were used to identify relevant papers on PTX3 as a biological marker in critically ill patients in the medical literature. First, an electronic search in the PubMed database was performed. Searches were also performed using the Cochrane Library and the Cochrane Database of Systematic reviews. Second, reference lists of identified and selected papers were reviewed for studies not identified with our search. Searches were restricted to original studies in humans and written in English.

### 2.2. Keywords (Text Word)

The following keywords were used, alone or in combination, to identity relevant papers: (1) condition “critical care” or “intensive care”, (2) subject “human”, (3) test “pentraxin 3” or “PTX3” or “soluble pentraxin 3 receptor” or “soluble PTX3” or “soluble PTX3 receptor” or “Tumor necrosis factor-stimulated gene 14” or “Tumor necrosis factor-stimulated gene 14 receptor” or “TSG-14”, and (4) disease “systemic inflammatory response syndrome,” “sepsis,” “septic shock,” or “bacteremia”.

### 2.3. Study Selection

Titles and abstracts were reviewed, and papers that reported on studies of PTX3 in critically ill patients with systemic inflammation or infection were selected. Thus, only papers on studies of critically ill patients were included (i.e., studies of patients who were admitted to a hospital with systemic inflammation or infection or to an intensive care unit (ICU)). In case of uncertainty, the complete paper was obtained and evaluated. Inclusion of papers was not restricted by methodological quality or any other critically appraisal criteria other than the criteria formulated for data extraction.

### 2.4. Quality Assessment

The quality of the included studies was evaluated by applying the 25-item criteria developed by the Standards for Reporting of Diagnostic Accuracy (STARD) committee [16, 17]. The maximum quality score that could be given to a study was 25 points over five categories. For each category, results were derived from consensus among all reviewers.

### 2.5. Data Extraction

Manuscripts were criticized along the following four subjects: (1) PTX3 is a useful diagnostic marker in detection of infection, (2) PTX3 is a useful prognostic marker in patients with SIRS, sepsis, or bacteremia, (3) systemic concentrations of PTX3 correlate with disease severity scores and markers of organ failure in these patients, and (4) how do systemic levels of PTX3 respond to initiation of treatment?

## 3. Results

### 3.1. Search Results

The search performed in April 2014 in the PubMed database revealed eleven papers as original studies [15, 18–27]. The Cochrane Library and the Cochrane Database of Systematic reviews revealed no reviews or meta-analyses of PTX3 in patients. Quality evaluation of the included studies using the STARD checklist is presented in Table 1.

Study populations in the retrieved studies comprised patients with SIRS, sepsis and/or septic shock, and bacteremia. Importantly, definitions for diagnoses varied among the studies.  Table 2 presents the criteria used for diagnosis of each study.

Seven studies evaluated the diagnostic value of PTX3 [15, 19, 21–26]; eleven studies investigated the prognostic value of PTX3 [15, 18–27]. Six studies correlated systemic levels of PTX3 to disease severity scores [15, 18, 20, 22, 24, 27]; four studies investigated changes of systemic levels of PTX3 after initiation of treatment [20–22, 27].

### 3.2. Diagnostic Value of PTX3

Systemic levels of PTX3 were significantly higher in critically ill patients, compared to healthy controls [15, 20, 21, 24, 27]. Higher levels of PTX3 were associated with the development of sepsis, severe sepsis, and septic shock [15, 18, 20, 21, 24–26]. Moreover, the area under the curve (AUC) of 0.922 (cut-off value 16.0 ng/mL, sensitivity 89.1%, and specificity 85%) to discriminate between healthy controls and SIRS was found [24]. Also, in patients with suspected infection admitted to the emergency room, the AUC for prediction of severe sepsis between day 0 and day 28 was 0.73 for PTX3 (cut-off value 14.1 ng/m, sensitivity 63%, specificity 80%) [25]. However, in patients who fulfilled at least two criteria for SIRS, using Dunn's posttest, it was revealed that only the SIRS group differed significantly from the other groups, while no significant difference was observed between the sepsis, severe sepsis, and septic shock groups.

Systemic levels of PTX3 were significantly higher in patients with blood culture-positive bacteremia compared to healthy controls [21] and blood culture negative samples [18, 24]. High levels of PTX3 were associated bacteremia [21, 23]. In febrile patients at the emergency department, the AUC in the prediction of bloodstream infection was 0.71, at a cut-off level for 16.1 ng/mL with 76% sensitivity and 61% specificity [21]. Finally, the PTX3 threshold of >16.43 ng/mL provided a specificity of 74.0% and a sensitivity of 68.6% for the diagnosis of ventilator-associated pneumonia (VAP) [26].

However, levels of PTX3 in patients with gram-positive bacteremia did not appear to be different from those with gram-negative bacteremia [21]. Also, the values did not differ significantly between patients with infection caused by different culprit organisms (i.e.,* Staphylococcus aureus*,* Streptococcus pneumoniae*, *β*-hemolytic Streptococcaceae, and* Escherichia coli*) [22]. But levels of PTX3 have been shown to be the highest in patients with urinary tract infections than in those with pulmonary infections and abdominal infection and other infections [20].

PTX3 values showed a positive correlation with other frequently used biological markers that can be measured easily with commercially available kits, including CRP, IL-6, procalcitonin (PCT), and calcitonin precursors (CTpr) [15, 24, 25]. In the case of sepsis patients, both PTX3 and high PCT seemed to be independent predictors of severe sepsis while CRP did not [25]. But the study of patients with SIRS showed that PTX3 and CRP had similar performance in discriminating between SIRS and sepsis [24]. However, in critically ill patients, PTX3 was no better than CTpr as a diagnostic index of infection and sepsis [15].

### 3.3. Prognostic Value of PTX3

Systemic levels of PTX3 were significantly high in critically ill patients with fatal outcomes compared to patients who survived critical illness [15, 18, 20, 23–26]. In patients with bacteremia, the maximum PTX3 values on days 1–4 were markedly higher in nonsurvivors compared to survivors (44.8 versus 6.4 ng/mL, *P* < 0.001) and the AUC in the prediction of case fatality was 0.82 (cut-off values 15 ng/mL, sensitivity 72%, and specificity 81%) [22]. Similarly, the AUC in emergency room patients with suspected infection was 0.69 (cut-off value 7.7 ng/mL, sensitivity 70%, specificity 63%) in predicting case fatality on day 28 [25]. Of note, the rate of positive blood cultures was 8.3% and case fatality by day 28 after admission 6.7%. In addition, SIRS patients with high levels of PTX3 at admission did have a higher 90-day mortality rate than patients with the 25% lowest levels (Cox regression analysis; hazard ratio 3.0; *P* = 0.0009) [24]. In patients with severe sepsis or septic shock, a value of day 5 : day 1 PTX3 of 0.76 predicted death with 64% specificity and 72% sensitivity (positive likelihood ratio 1.98). Every 0.01 increase in day 5 : day 1 PTX3 levels increased the probability of death by 3.7% (95% confidence interval 1.2–6.3%) [20].

As a single biological marker, PTX3 was superior in predicting mortality compared to other frequently used biological markers, including CRP, IL-6, and TNF-*α* (Table 3) [15, 18, 20, 22–26]. The ability of PTX3 to predict mortality was poor compared to the conventional simplified acute physiology score (SAPS) II [15].

### 3.4. Correlation of PTX3 with the Severity of Disease

In patients with suspected infection, a high PTX3 level (≥14.1 ng/mL; optimal cut-off value for severe sepsis) was associated with several endpoints reflecting the severity of the disease (need for ICU stay, hypotension, and acute renal insufficiency) [25]. Similar associations were described for blood culture-positive bacteremic patients [22]. In patients with meningococcal disease, high PTX3 and low CRP concentration at admission discriminated between presence and absence of shock [19]. In patients with community-acquired pneumonia (CAP), the plasma concentration of PTX3, but not CRP, was correlated with the severity of CAP based on the pneumonia severity index (PSI), CURB-65, Acute Physiology and Chronic Health Evaluation (APACHE) II scores, and the length of hospital stay [27].

In critically ill patients admitted to the medical ICU, systemic levels of PTX3 also correlated with disease severity, as assessed by the APACHE II and SAPS II scores or sequential organ failure assessment (SOFA) score [15, 18, 20, 24]. PTX3 levels were shown to better correlate with severity of the disease and organ dysfunctions than other measured mediators (i.e., TNF*α*, IL-6, and CRP) [20]. Of note, in patients with severe sepsis or septic shock, Day1 PTX3 was correlated with platelet count and prothrombin fragments 1 + 2 concentration and with plasminogen activator inhibitor-1 (PAI-1) activity and concentration [20]. Moreover, in patients admitted to the intensive-/medium care unit, the levels of PTX3 were high in comparison to levels of patients referred to the ward. PTX3 was associated with duration of hospital stay and acute congestive heart failure [21].

### 3.5. Systemic Levels of PTX3 after Initiation of Treatment

Six studies evaluated levels of PTX3 after the initiation of treatment [15, 18, 20–22, 27]. Systemic levels of PTX3 remained peaked during the first hours after hospital admission and were high in the acute phase and decreased after antibiotic treatment [18, 22, 27]. At admission, levels of PTX3 in febrile patients were significantly elevated in comparison to samples collected after full recovery at a one-month follow-up visit and correlated with the total duration of antibiotic treatment [21]. PTX3 remained elevated in the presence of unremitting disease [15]. PTX3 levels did not differ between the tight glycemic control strategy and conventional glycemic control strategy group for the first 5 days from enrollment (*P* = 0.221) [20].

### 3.6. Factors That Influence Levels of PTX3 and/or Its Performance

Some studies showed that age of patients and underlying diseases affected levels of PTX3 and/or its performance. Studies that investigated systemic levels of PTX3 in relation to age are contradictory [22–25]. Two studies found no correlation between systemic levels of PTX3 on day 0 and age, sex, or comorbidities [22, 23]. However, two other studies suggested higher levels of PTX3 in elderly patients [24, 25]. In patients with bacteremia, levels of PTX3 were high in patients with chronic alcohol abuse compared to patients without a history of alcohol abuse, while there was no difference between groups of patients stratified by other chronic conditions, age, sex, or causative organism [22]. Interestingly, serum concentrations of PTX3 did not differ between sexes nor by whether the patients were admitted with a surgical or nonsurgical diagnosis [24]. PTX3 values were significantly high in patients with obesity (body mass index (BMI) ≥ 30), cardiovascular diseases, and continuous systemic cortisone treatment (daily dose over 10 mg oral prednisolone) compared to those without these risk factors [25].

In one study, highly specific monoclonal antibodies against human PTX3 were produced and used to develop the new sandwich ELISA system. With this, PTX3 was detectable in serum at concentrations down to 0.3 ng/mL. Moreover, the established assay was very stable and capable of measuring PTX3 exposed to consecutive freezing thawing cycles. In addition, a strength of this new assay was the very high correlation with a commercially available PTX3 assay (rho 0.8, *P* < 0.001) [24].

## 4. Discussion

This systematic review shows that although systemic levels of PTX3 are elevated in more severe forms of sepsis and bacteremia, its diagnostic value is low, as PTX3 is a nonspecific marker of inflammation. Systemic levels of PTX3, however, do have prognostic value, with higher levels being associated with poor outcome. Systemic levels of PTX3 correlate positively with severity-of-disease classification scores and peaked earlier than CRP. Systemic levels of PTX3 also correlate positively with several markers of organ dysfunction.

The mortality rate of SIRS, sepsis, severe sepsis, and septic shock is high, ranging from approximately 10% in SIRS to 60% in septic shock [28]. While identification and treatment of sepsis has led to a decrease in mortality, the incidence continues to increase resulting in a still larger number of deaths annually [29]. Worldwide, sepsis is thus the major cause of death in intensive care units and therefore an area of concern [30]. In order to improve the survival of patients with SIRS and sepsis, it is essential to identify the individuals at high risk. One approach for this identification is constantly evaluating reliable biological markers. Traditional markers such as white blood cell count, fever, and CRP levels are not reliable for assessing disease severity and mortality risk [31, 32]. Procalcitonin seems to be an improvement on these markers, but is not ideal [33–35]. Although PCT has repeatedly shown prognostic value in critically ill patients, the value of a single level on admission is limited [36]. The compiled data in this paper suggest that PTX3 has superior prognostic value compared to commonly used biological markers, including CRP. Moreover, PTX3 levels were high in the acute phase and reached the maximal point earlier than CRP. PTX3 may be used as an early biomarker in sepsis patients.

Pentraxin 3 is the prototype of the long pentraxin family [37, 38]. It differs from CRP in terms of gene organization and localization, ligand recognition, producing cells, and inducing signals [39–41]. CRP is produced in the liver, whereas PTX3 is an inflammatory mediator produced by various cells in peripheral tissues. PTX3 values in healthy subjects are low (i.e., <2 ng/mL). PTX3 can be rapidly produced and released by mononuclear phagocytes, neutrophils, fibroblasts, and epithelial and endothelial cells in response to primary inflammatory signals (e.g., IL-1 and TNF-*α*) [42]. It plays an important role in the early phase of inflammation: it recognizes microbial moieties, activates the classical pathway of complement, and facilitates recognition by macrophages and dendritic cells [43]. PTX3 has an important role in regulating the innate immune response by contributing to the opsonization and clearance of apoptotic or necrotic cells [44].

Although PTX3 alone was not performed as well as the SAPS II score, this does not necessarily preclude its use in prognostication. APACHE II score, SAPS II, SOFA score, and other scoring systems estimating the risk of mortality have become increasingly popular in the field of research with critically ill patients over the last decades. However, in clinical practice, these scoring systems have important limitations. Data collection requires multiple laboratory measurements and the computation of multiple variables and is labor intensive and expensive [45–47]. Therefore, the application of these scoring systems may be limited, particularly when health care is subject to financial constraint. PTX3 may have other important advantages. Only one blood sample instead of multiple clinical and laboratory measurements are needed. Measurement of PTX3 can be performed using a simple ELISA. In addition, PTX3 is stable in plasma samples subjected to repeated freeze-thaw procedures [24], increasing its practicality as a practical biological marker. Thus, based on the findings that systemic levels of PTX3 are a strong and robust marker of mortality risk, one could speculate that PTX3 will eventually serve as a quick, technically simple, and inexpensive alternative to the current sophisticated severity-of-disease classification systems. Future studies are needed to address this hypothesis.

Notably, systemic levels of PTX3 remain elevated in the acute phase and decreased on recovery. Therefore, the use of PTX3 as a biological marker for guiding therapy is possible. However, studies addressing this issue are lacking. Importantly, the type of assay used to measure PTX3 and age and presence or absence of underlying diseases all influence PTX3 levels. The difference performance between different assays can be explained by the fact that the new sandwich ELISA assay uses highly specific monoclonal antibodies [24]. With the prognostic value of PTX3 in combination with other markers, this might compensate for the slightly impaired performance.

The finding that age as well as underlying disease influences the systemic level of PTX3 is of limited relevance as both age and underlying diseases are known to increase mortality risk in critically ill patients [48, 49]. Systemic levels of PTX3 remained independently prognostic for mortality after adjusting for age and/or underlying diseases [20, 25–27]. As with other markers, such as CRP and PCT, experience will eventually dictate the value of PTX3 levels in diverse clinical situations.

Of interest, PTX3 is not only present in human plasma or serum but can also be found in other body fluids, including urine [50, 51], cerebrospinal fluid [52], pleural [53, 54], amniotic fluid [55], and joint fluid [56, 57]. The number of studies investigating the value of PTX3 in body fluids other than plasma or serum, however, is very limited. It would be interesting to evaluate the value of local levels of PTX3 in other body fluids, that is, in bronchoalveolar lavage fluid of patients with frequent pulmonary complications, such as acute lung injury [18].

Finally, this review has limitations. The most common limitation of any systematic review is publication bias. Unpublished materials were not found and thus not used. However, the results from these relatively small studies need to be further validated in larger, multicenter trials before this approach can be recommended. Moreover, the heterogeneous nature of the current studies prevents any meta-analytic technique to derive an optimal range of values for prognostication.

## 5. Conclusions

The studies that have evaluated PTX3 levels vary in the types of patient populations studied, the basal conditions of the patients, and the methods used to measure PTX3. PTX3 in diagnosing sepsis does not appear to be superior to other biomarkers, like CRP and PCT. PTX3 seems a promising prognostic marker in critically ill patients. Currently, studies are limited to the predictive potential to estimate the mortality risk in observational designs. The monitoring of PTX3 levels during therapy needs further study to determine whether this biomarker could be of use in guiding therapeutic decisions.

## Figures and Tables

**Table 1:** Quality evaluation of the included studies using the Standard for Reporting of Diagnostic Accuracy (STARD) checklist [16, 17].

Item^a^	Title/abstract/Key words	Introduction	Methods											Results											Discussion	Totle
			Participants				Test methods					Statistical methods		Participants			Test results				Estimates			
	1	2	3	4	5	6	7	8	9	10	11	12	13	14	15	16	17	18	19	20	21	22	23	24	25	25
Muller et al., 2001 [15]	1	1	1	1	1	1	1	1	1	0	0	1	0	1	0	1	1	0	1	0	1	0	1	0	1	17
Mauri et al., 2008 [18]	1	1	1	1	1	1	1	1	1	0	0	1	1	1	1	1	1	1	1	0	1	0	1	0	1	20
Sprong et al., 2009 [19]	1	1	0	1	0	1	0	1	1	0	0	1	0	1	1	1	1	0	1	0	1	0	1	0	1	15
Mauri et al., 2010 [20]	1	1	1	1	1	1	1	1	1	0	0	1	0	1	1	0	1	1	0	0	1	0	1	0	1	17
de Kruif et al., 2010 [21]	0	1	1	1	1	1	1	1	1	0	1	1	0	1	1	1	1	1	1	0	1	0	0	0	1	18
Huttunen et al., 2011 [22]	0	1	1	1	1	1	1	1	1	0	1	1	1	1	1	0	1	1	1	0	1	0	1	0	1	19
Vänskä et al., 2011 [23]	0	1	1	1	1	1	1	1	0	0	1	1	0	1	1	1	1	0	1	0	0	0	1	0	1	16
Bastrup-Birk et al., 2013 [24]	1	1	1	1	1	1	1	1	0	0	0	1	0	1	1	1	1	0	1	0	1	0	1	0	1	17
Uusitalo-Seppälä et al., 2013 [25]	1	1	1	1	1	1	1	1	1	0	0	1	0	1	1	1	1	1	1	0	1	0	1	0	1	19
Lin et al., 2013 [26]	1	1	1	1	1	1	1	1	1	0	0	1	1	1	1	1	1	1	1	0	1	0	0	0	1	19
Kao et al., 2013 [27]	1	1	1	1	1	1	1	1	1	0	0	1	0	1	1	1	1	1	1	0	1	1	0	0	1	19

^a^For each item, results are derived from consensus among all reviewers as the number of items from the checklist present in the original article.

**Table 2:** Patients characteristics of the included studies.

First author	References	Criteria used for diagnosis	Diagnosis on admission as described by the authors (no. of patients)	Period
Muller	[15]	SIRS, sepsis, severe sepsis, and septic shock criteria	Sepsis, severe sepsis, or septic shock (33) SIRS (68)	September 1996 to June 1997

Mauri	[18]	ALI/ARDS criteria SIRS, septic, severely septic or septic shock criteria	ALI/ARDS (21) Nonseptic (3), SIRS (4), septic (0), severely septic (5), septic shock (9)	January 2005 to October 2006

Sprong	[19]	Meningococcal disease	meningococcal disease (26)	1994 to 2004

Mauri	[20]	Severe sepsis or septic shock criteria	Severe sepsis (18), septic shock (72)	December 2004 to March 2007

de Kruif	[21]	Fever criteria, bacteremia criteria	Febrile patients (211), bacteremia (35)	April 2004 to October 2006, one month follow-up after enrollment

Huttunen	[22]	Blood culture positive for *S. aureus*, *S. pneumoniae*, *β*-hemolytic *Streptococcus* or *E. coli*	Bacteremia (132)	June 1999 to February 2004

Vänskä	[23]	Neutropenic fever and septic chock criteria	Neutropenic fever (100), septic shock (5)	December 2006 to December 2009

Bastrup-Birk	[24]	SIRS criteria	SIRS (261)	Undefined, median follow up time 873 (range, 0–1458) days

Uusitalo-Seppälä	[25]	Suspected infection : Bacterial infection, SIRS, Sepsis and Severe sepsis criteria	Suspected infection (537) : Bacterial infection (67), SIRS (54), Sepsis (308), severe sepsis (49)	2004 to 2005, 14 months, one year follow-up after enrollment

Lin	[26]	VAP criteria	VAP (86)	January 2010 to December 2011, 28 days follow-up after enrollment

Kao	[27]	CAP criteria	CAP (61)	January 2009 to December 2009

**Table 3:** Prognostic value of PTX3 to predict mortality as compared to other biological markers and disease severity scores.

Ability to predict	Parameter	AUC (95% CI)	Cutoff	Sensitivity (%)	Specificity (%)	References
General intensive care unit population						
	PTX3	0.63	—	—	—	
	IL-6	0.72	—	—	—	
ICU mortality	CRP	0.55	—	—	—	[15]
	CTpr	0.61	—	—	—	
	SAPSII	0.76	—	—	—	
90-Day mortality	PTX3 (day 5 : day 1)	0.73	0.76	64	72	[20]
	CRP (day 5 : day 1)	0.58	—	—	—	
	IL-6 (day 5 : day 1)	0.63	—	—	—	
	IF-*α* (day 5 : day 1)	0.61	—	—	—	

Patients with infectious diseases						
	PTX3	0.69 (CI 0.58–0.79)	7.7 ng/mL	70	63	
28-Day mortality	PCT	0.65 (CI 0.57–0.74)	0.19 ng/mL	82	63	[25]
	CRP	0.50 (CI 0.38–0.62)	—	—	—	

Bacteremic patients						
30-Day mortality	PTX3 (the maximum value on days 1 to 4)	0.82 (CI 0.73–0.91)	15 ng/mL	72	81	[22]
	CRP (the maximum value on days 1 to 4)	0.61 (CI 0.49–0.73)	—	—	—	
	PTX3	0.781 ± 0.065	>44.45 ng/mL	64.7	88.4	
28-Day mortality	CRP	0.635 ± 0.071	>135.7	64.7	62.3	[26]
	PTX3 + CRP	0.863 ± 0.041	—	—	—	
